# Notes and Notices

**Published:** 1885-07

**Authors:** 


### NOTES AND NOTICES.

Ocular Surgery.—Dr. Landolt, of Paris, will commence this summer a course of practical lectures on operations on the eye. Should there be a sufficient number of American medical men who may wish to attend regularly, the Professor will have much pleasure in forming a separate class for them, at which the lectures will be delivered in English. For further particulars, please address Dr. Landolt, 4 Rue Volney, Paris, France, or Dr. John H. Payne, 415 Columbus Avenue, Boston.

Case for Consultation.—I have a case of a man thirty-five years old, engaged in the boot and shoe business. His finger nails are being pressed away from the fingers (and thumbs) by a growth. It seems like flesh, causes no pain, only looks bad. The nail dies as it is pressed off, sometimes even to the quick. He says he has known others engaged in the leather business to be so afflicted. I have never seen such a case before. Can you give me a name or treatment for it? If not, will you put it to your readers? C. G. W.

The Delinquents.—It is evident from the following clipping that medical journals are not the only sufferers from delinquent subscribers:

The St. Louis *Presbyterian*, in trying to explain to its subscribers why it sends out bills so promptly and persistently, says: "We presume that some people think newspaper men are persistent duns. Let a farmer place himself in the same position, and see if he would do the same. Suppose that he raises several thousand bushels of corn and his neighbor should come and buy a bushel and say, 'I will pay you the amount in a few days.' As the farmer doesn't want to be small about the matter, he says, 'All right.' Another comes in the same way, until the whole of his corn is gone, and not one of the purchasers concerns himself about it, because it is a small amount that he owes the farmer. He does not realize that the farmer has frittered away his large crop of corn, and that its value is due in thousands of little driblets, and that he is seriously embarrassed in his business because his debtors treat it as a little matter."

Subscriptions to the new Repertory of Characteristics continue to come. Still the required number is not yet received. All physicians should subscribe at once. As soon as the required number is obtained, we will at once start the presses and turn out the work as quickly as possible.

